# Does tuberculosis screening improve individual outcomes? A systematic review

**DOI:** 10.1016/j.eclinm.2021.101127

**Published:** 2021-09-22

**Authors:** L Telisinghe, M Ruperez, M Amofa-Sekyi, L Mwenge, T Mainga, R Kumar, M Hassan, L.H Chaisson, F Naufal, A.E Shapiro, J.E Golub, C Miller, E.L Corbett, R.M Burke, P MacPherson, R.J Hayes, V Bond, C Daneshvar, E Klinkenberg, H.M Ayles

**Affiliations:** a London School of Hygiene and Tropical Medicine, London, UK; b Zambart, University of Zambia School of Public Health, Ridgeway, Zambia; c University Hospitals Plymouth NHS Trust, UK; d Chest Diseases Department, Faculty of Medicine, Alexandria University, Egypt; e Division of Infectious Diseases, Department of Medicine, University of Illinois at Chicago, Chicago, USA; f Wilmer Eye Institute, Johns Hopkins University, Baltimore, USA; g Departments of Global Health and Medicine, University of Washington, Seattle, USA; h Johns Hopkins University School of Medicine, Center for Tuberculosis Research, Baltimore, USA; i Global TB programme, World Health Organization, Geneva, Switzerland; j Malawi-Liverpool-Wellcome Trust Clinical Research Programme, Blantyre, Malawi; k Liverpool School of Tropical Medicine, Liverpool, UK; l Department of Global Health and Amsterdam Institute for Global Health and Development, Amsterdam University Medical Centres, Amsterdam, The Netherlands

**Keywords:** Screening, Active case-finding, Enhanced case-finding, Individual effects, Disease severity, Treatment outcomes, Mortality, Case fatality, Economic consequences, Patient costs, Catastrophic costs

## Abstract

**Background:**

To determine if tuberculosis (TB) screening improves patient outcomes, we conducted two systematic reviews to investigate the effect of TB screening on diagnosis, treatment outcomes, deaths (clinical review assessing 23 outcome indicators); and patient costs (economic review).

**Methods:**

Pubmed, EMBASE, Scopus and the Cochrane Library were searched between 1/1/1980-13/4/2020 (clinical review) and 1/1/2010-14/8/2020 (economic review). As studies were heterogeneous, data synthesis was narrative.

**Findings:**

Clinical review: of 27,270 articles, 18 (n=3 trials) were eligible. Nine involved general populations. Compared to passive case finding (PCF), studies showed lower smear grade (n=2/3) and time to diagnosis (n=2/3); higher pre-treatment losses to follow-up (screened 23% and 29% vs PCF 15% and 14%; n=2/2); and similar treatment success (range 68-81%; n=4) and case fatality (range 3-11%; n=5) in the screened group. Nine reported on risk groups. Compared to PCF, studies showed lower smear positivity among those culture-confirmed (n=3/4) and time to diagnosis (n=2/2); and similar (range 80-90%; n=2/2) treatment success in the screened group. Case fatality was lower in n=2/3 observational studies; both reported on established screening programmes. A neonatal trial and post-hoc analysis of a household contacts trial found screening was associated with lower all-cause mortality. Economic review: From 2841 articles, six observational studies were eligible. Total costs (n=6) and catastrophic cost prevalence (n=4; range screened 9-45% vs PCF 12-61%) was lower among those screened.

**Interpretation:**

We found very limited patient outcome data. Collecting and reporting this data must be prioritised to inform policy and practice.

**Funding:**

WHO and EDCTP.

**Research in context**

**Evidence before this study**

Tuberculosis (TB) remains a leading infectious cause of death worldwide, and therefore improving access to diagnosis and treatment, closing the case-detection gap and improving patient outcomes is a priority. In 2019, a MEDLINE and EMBASE search for English language articles on TB screening identified a systematic review. Synthesising data published between 1/1/1980-13/10/2010, it found little evidence that TB screening benefited individuals screened; patient costs were not assessed.

**Added value of this study**

Synthesising evidence between 1980-2020, our systematic review investigating the effects of TB screening on patient outcomes, found 24 articles (including three trials) from 12 countries. The limited available data suggests that compared to passive case finding, TB screening may be associated with less severe disease; decreased time to diagnosis/first contact with health services; decreased deaths (among risk groups alone); decreased patient costs; and higher pre-treatment losses to follow-up. There was no difference in treatment success between screened and passive case finding groups.

**Implications of all the available evidence**

With World Health Organization targets to END-TB calling for decreases in TB deaths, incidence and catastrophic costs, countries have renewed their interest in TB screening, to find, test and treat “the missing millions”. We found very limited data on the individual effects of TB screening. Routine/research programme implementation must be combined with rigorous data collection and analysis of critical patient outcomes that allows the benefits and harms of TB screening to be characterised.

Alt-text: Unlabelled box

## 1 Introduction

Despite effective, curative treatment, tuberculosis (TB) is a leading infectious cause of death worldwide [1]. In most TB-endemic settings, standard case-detection through routine services (passive case-finding [PCF]), is the mainstay of access to TB diagnosis and treatment [2,3]. This may be augmented by facility-based TB screening in specific high-risk populations, such as people living with HIV/AIDS. But these measures alone do not identify the substantial burden of undiagnosed TB in these settings, or effectively reach the poor and vulnerable who face barriers to seeking health care [[3], [4], [5]]. In 2019, ∼3 million TB patients were either not diagnosed or not notified [1]. If untreated, TB is associated with high mortality and morbidity [6]. Therefore, closing the case-detection gap by improving access to TB diagnosis and treatment is a priority.

One strategy to address this is TB screening, which encompasses a wide range of activities aimed at detecting and treating TB patients earlier in their clinical course [4 ,5]. This should improve the individual's clinical outcomes, [4,5] a requirement for traditional screening programmes [7]. While infectious diseases screening can have both individual and population effects, [4] understanding whether screening benefits the individual is critical when considering if to screen. The costs borne by people seeking TB services and their households (patient costs) can be high, hindering diagnosis and treatment [8]. Such costs can exacerbate poverty, increasing the vulnerability of individuals, with further social and health consequences [9,10]. TB screening, by helping individuals navigate the TB care pathway, may also potentially decrease patient costs.

But evidence that TB screening improves clinical outcomes and reduces patient costs is lacking [4,11]. Therefore, we undertook two systematic reviews to determine if TB screening 1) identifies TB patients earlier in their clinical course; improves linkage-to-care; improves treatment outcomes; and decreases deaths (clinical review) and 2) decreases patient costs (economic review).

## 2 Methods

We undertook two systematic reviews to identify studies reporting the effect of TB screening on clinical outcomes and patient costs. These were conducted to inform World Health Organization (WHO) TB screening guideline development. The Population, Intervention, Comparison(s) and Outcomes were determined in collaboration with the guideline development group (GDG), consisting of a panel of experts in the field of TB. The methods followed standard procedures for undertaking systematic reviews [12] and grading evidence quality [13].

### 2.1 Study populations, interventions, outcomes and definitions

Studies conducted in any population group were considered. Screening was defined as any provider-initiated intervention including 1) using health information/education to encourage appropriate health-seeking behaviours, with or without increasing access to diagnostic services (enhanced case-finding [ECF]); and 2) systematic screening using any test/procedure (active case-finding in communities [ACF] and case-finding in health facilities). PCF, the comparator, was defined as the routine diagnosis of symptomatic TB patients self-presenting to health services.

We included 23 clinical outcome indicators (Table 1) for earlier diagnosis (e.g. smear grade, body mass index), linkage-to-care (e.g. pre-treatment loss to follow-up [LTFU]), treatment outcome (e.g. success) and death (e.g. case fatality, mortality). These outcomes were all rated as critical or very important by the GDG. Clinical outcomes were assessed among bacteriologically-confirmed TB patients (culture, Xpert MTB/RIF or smear positive). Treatment success was defined as cured and treatment completed (without microbiological evidence of cure) [14]. Pre-treatment LTFU was defined as LTFU between diagnosis and treatment start. Patient cost input data (Table 1) were broadly categorised as direct medical (e.g. hospitalisation costs), direct non-medical (e.g. transportation) and indirect (e.g. lost productivity). Patient costs were assessed among all TB patients (bacteriologically-confirmed and clinically diagnosed). Catastrophic cost was defined as total costs for seeking TB care >20% of the annual household income [1].

**Table 1:** Clinical outcomes and patient costs* for the clinical and economic review

Clinical outcomes for clinical review			
Outcome category		Outcome indicator	
		Sought	Identified
Earlier diagnosis	Disease severity at diagnosis - microbiology	smear positivity among bacteriologically-confirmed TB patients; smear grade; Xpert cycle threshold values; culture grade/colonies; time to culture positivity	smear positivity among bacteriologically-confirmed TB patients; smear grade
	Disease severity at diagnosis - radiology	CXR severity score/grading	-
	Disease severity at diagnosis - anthropometric	body mass index	-
Earlier diagnosis and linkage to care	Time to first contact with health services	duration from start of symptoms to first contact with health services	duration from start of symptoms to first contact with health services
	Time to diagnosis	duration from start of symptoms to diagnosis	duration from start of symptoms to diagnosis
	Time to treatment start	duration from start of symptoms to treatment start; time between diagnosis and treatment start	duration from start of symptoms to treatment start; time between diagnosis and treatment start
	Pre-treatment loss to follow-up	lost to follow-up between diagnosis and treatment start	lost to follow-up between diagnosis and treatment start
Treatment	Treatment outcomes at treatment end	treatment success (cure and completion); lost to follow-up	treatment success (cure and completion); lost to follow-up
	Disease outcome at treatment end - morbidity	body mass index; lung function test results; TB recurrence	-
Deaths	Mortality among screened and unscreened groups	all-cause mortality; TB-specific mortality	all-cause mortality
	Case fatality among diagnosed TB patients	all-cause case fatality; TB-specific case fatality	-
	Case fatality among treated TB patients	all-cause case fatality; TB-specific case fatality	all-cause case fatality; TB-specific case fatality

Patient costs* for economic review			
Outcome category		Outcome - cost input	

Pre-diagnosis	Costs before TB diagnosis	Direct medical - consultation/administration fees; drugs (TB, other); hospitalisation; laboratory investigations, radiology investigations, other investigations Direct non-medical costs - transport, food, accommodation, nutritional supplements, childcare Indirect - productivity loss	
Diagnosis	Costs during TB diagnosis		
Pre-treatment	Costs before TB treatment May include pre-diagnosis and diagnosis costs		
Treatment	Costs during TB treatment		
Entire illness period	Costs during the illness period reported in the study		
Catastrophic cost	Prevalence	proportion of total cost for TB care >20% of annual household income	

*costs incurred by TB patients and their households

### 2.2 Search strategy

**Figure 1:**
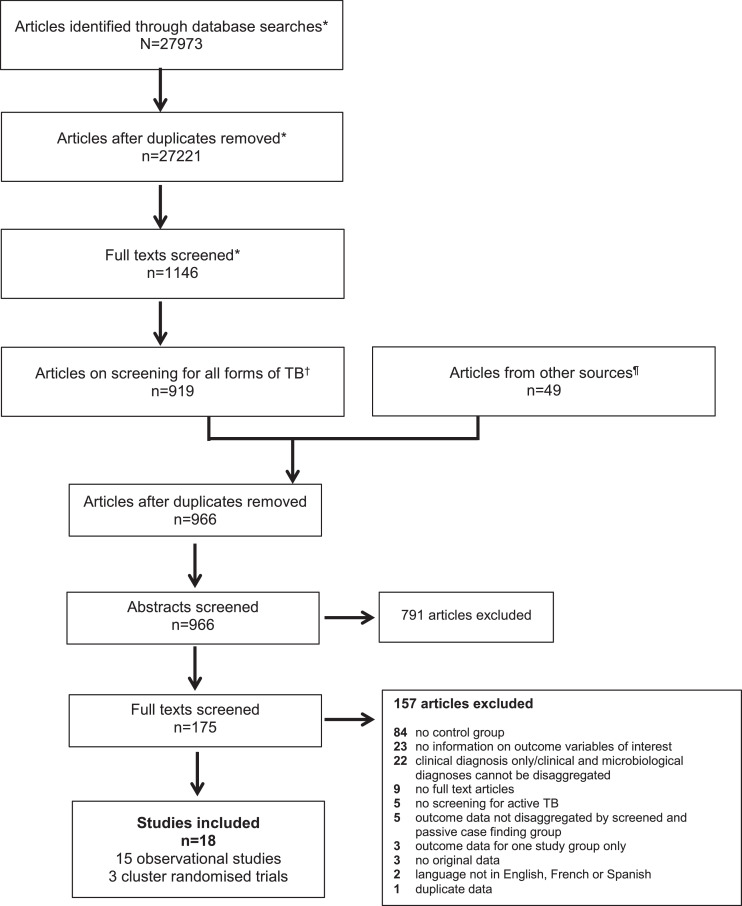
Study selection process - flow diagram of number of original research articles considered for the clinical review. The clinical review was nested within a systematic review to determine the number needed to screen to detect a TB patient in any population. *represents the study selection process for the number needed to screen review. ^†^The starting point of the clinical review, which is reported in this manuscript. ^¶^Previous systematic review by Kranzer et al 2013, authors and bibliography searches.

Clinical review: we updated the systematic review conducted by Kranzer 2013, [11] which covered the period 1/1/1980-13/10/2010 (Figure 1). Articles addressing the research questions from the Kranzer 2013 review were also included in our review. Our update used the same methods as Kranzer 2013; the search was nested within a systematic review to determine the number needed to screen to detect a TB patient in any population [15]. For the number needed to screen review, Pubmed, EMBASE, Scopus and the Cochrane Library were searched from 1/11/2010-13/4/2020. Subject headings and key words covered the concepts of TB and screening (Appendix 1). The title and abstract screens were broad; articles needed to be original research on TB screening. Full text screens determined eligibility. Articles from the number needed to screen review reporting on screening for all forms of TB were assessed for eligibility for our review.

Economic review: Medline, EMBASE, Scopus and the Cochrane Library were searched from 1/1/2010-14/8/2020. Subject headings and key words covered the concepts of 1) TB; 2) screening; and 3) economic evaluations or economic/financial analysis (Appendix 1). The Global Health Cost Consortium Unit Cost Study Repository was also searched for additional articles [16].

For both reviews, bibliographies of identified studies were searched, and authors contacted for additional data if needed.

### 2.3 Eligibility criteria

Only articles in English, French and Spanish were included. Both (quasi-)randomised controlled trials (RCTs) and observational studies with screened and PCF groups were eligible. Studies comparing two different screening strategies or where screening and PCF occurred in different populations (e.g. screened miners and PCF in the general population) were excluded. Observational studies not disaggregating data by screened and PCF groups were excluded. RCTs (individual and cluster [CRTs]) comparing treatment, death and cost outcomes by randomised arm were eligible, as this design can mitigate biases inherent in observational screening studies. For the clinical review, household contact screening studies where index cases formed the PCF group and household contacts the screened group were excluded as individuals from the same households are clustered.

### 2.4 Study selection, data extraction and risk of bias assessment

Study selection, data extraction and risk of bias assessments were undertaken by two independent reviewers (LT, MR, MAS, MH and CD conducted the clinical review and LM, and EK conducted the economic review). Disagreements were resolved through discussion or, if required, consultation with a third reviewer.

For the clinical review, abstracts of articles were searched to shortlist studies with a control population (parallel or before-after design). For the economic review, articles were initially shortlisted based on the title and abstract. For both reviews, inclusion was based on full text review of shortlisted articles.

Data were extracted into case report forms. Variables extracted included study design, population, calendar period, screening strategy, PCF algorithm, TB case definition, participant numbers and outcome data. Methodological quality of cross-sectional studies was assessed across four domains; valid participant selection, valid exposure ascertainment, valid outcome ascertainment, and adequate control for confounders [13]. Quality assessment of CRTs was undertaken using the Cochrane Risk of Bias tool [17,18]. For economic studies the Consolidated Health Economic Evaluation Reporting Standards (CHEERS) statement was used [19].

### 2.5 Data synthesis and analysis

Due to the heterogeneity of included studies (populations, screening tools, effect estimates, etc), data synthesis for both reviews was narrative. For treatment success and on-treatment case fatality calculations, we only included cured, treatment completed, death, treatment failure, LTFU, and not evaluated (including transferred out) in the denominator; other outcomes reported, such as still on treatment, were excluded. Smear grade was recategorized, with grades scanty/1+/2+ combined to reflect lower grades (and less extensive disease) and 3+ reflecting higher grades (and more extensive disease). A sensitivity analysis was conducted recategorizing smear grades scanty/1+ as lower grade and 2+/3+ as higher grade. Where proportions were reported, 95% confidence intervals (95%CI) were calculated using Stata version 15 (StataCorp).

### 2.6 Role of the funding source

The WHO commissioned this work to inform TB screening guideline development. The WHO had no role in the conduct of the study or writing the report. The corresponding and last author had access to all data and final responsibility for the decision to submit for publication.

## 3 Results

### 3.1 Clinical review

From 27,270 articles, 18 were eligible [[20], [21], [22], [23], [24], [25], [26], [27], [28], [29], [30], [31], [32], [33], [34], [35], [36], [37]] (Figure 1 and Table 2); seven were not reported in the previous review [20,[29], [30], [31], [32],^36^,^37^]. We only identified n=12/23 (52%) of the outcome indicators sought (Table 1); no studies reported on the remainder. All studies reported on smear and/or culture positive TB (Table 2); no studies reported on Xpert MTB/RIF positive TB.

**Table 2:** Characteristics of studies included in the clinical review (N=18) and economic review (N=6)

First author and Location	Population	Study years	Screening: strategy and tools	TB case definition	Sample/cohort*		Outcomes *OR* Details of costing studies and costs collected
					Screen	PCF	
Clinical review – general population observational studies							
Abdurrahman 2016 Abuja, Nigeria	Urban including slums	2010-2014	ACF: One off community health worker house-to-house symptom screen. Sputum collected for smear if symptoms.	Smear + Adult ≥18 years	485	209	Smear grade Symptom duration at diagnosis
den Boon 2008 Cape Town, South Africa	2 suburbs	2002-2005	Prevalence survey: sputum smear and culture for all collected at health centres.	Smear or culture + Adult ≥15 years	27	473	Smear grade Treatment outcomes
Shargie 2006 Hadiya zone, Southern Ethiopia	Rural 1 district	2003	Prevalence survey: symptoms and/or on TB treatment. Sputum collected for smear if +.	Smear + Adult ≥15 years	13	24	Symptom duration at treatment start
Gopi 2005 Tiiruvallur South India	Rural and urban 1 sub-district	2001-2003	Prevalence survey: CXR and symptoms. Sputum collected for smear and culture if symptoms or abnormal CXR.	Smear + Adult ≥15 years	243	1049	Pre-treatment loss to follow-up
Balasubramanian 2004; Tiiruvallur South India	Rural and urban 1 sub-district	1998-2001	Prevalence survey: CXR and symptoms. Sputum collected for smear and culture if symptoms or abnormal CXR.	Smear + Adult ≥15 years	231	833	Pre-treatment loss to follow-up
Santha 2003 Tiiruvallur South India	Rural and urban 1 sub-district	1999-2000	Prevalence survey: CXR and symptoms. Sputum collected for smear and culture if symptoms or abnormal CXR.	Smear +	96	330	Smear grade Symptom duration at first contact with health services Treatment outcomes
Harper 1996 East Nepal	Rural 8 districts	1990-1993	Likely ECF (unclear): outreach TB camps (diagnostic services) lasting 2-4 days with pre-camp publicity in areas away from health posts, with high TB burden or where community requested services. If symptomatic sputum collected at camps. 45 camps over 3 years.	Smear + New TB	68	1306	Treatment outcomes
Cassels1982 East Nepal	Rural 1 district	1978-1980	ACF: one-off house-to-house symptom screen by vaccinators. Pots left for sputum collection if symptoms, with drop-off at designated centres within 20 minutes walking distance.	Smear +	111	159	Treatment outcomes

Clinical review – general population cluster randomised trials							
Shargie 2006 Hadiya Zone Southern Ethiopia	Rural 2 districts	2003-2004	ECF: x1/month for 12 months IEC activities by community promoters¶ encouraging those with symptoms to attend monthly diagnostic outreach clinic where sputum collected for smear.	Smear +	159	221	Treatment outcomes

Clinical review – risk groups observational studies							
Shewade 2019ʃ 18 districts in 7 states across India	Marginalised/vulnerable populationsŦ	2016-2017	ACF: one-off community volunteer house-to-house symptom screen. Referral for sputum smear if symptoms.	Smear + Adult ≥15 years	275	297	Smear grade Treatment outcomes
Shewade 2019ʃ 18 districts in 7 states across India	Marginalised/vulnerable populationsŦ	2016-2017	ACF: one-off community volunteer house-to-house symptom screen. Referral for sputum smear if symptoms.	Smear + Adult ≥15 years	234	231	Duration of symptoms to 1) first contact with health services; 2) diagnosis Time between diagnosis and treatment start Time between symptoms and treatment start
Paiao 2016 Mato Grosso do Sul state, Brazil	Prisoners in 12 prisons	2013-2014	ACF: x2 symptom screen (at baseline and 1 year later). Sputum collected if symptoms.	Culture + Adult ≥18 years	40	53	Smear positivity of culture confirmed TB patients
Story 2012 London, UK	Homeless people, drug users, asylum seekers, prisoners	2005-2010	ACF: mobile CXR screening programme. Screening in community settings where hard to reach people can be accessed (e.g. hostels, day centres, drug treatment services, prisons).	Culture + Age >15 years	23	146	Smear positivity of culture confirmed TB patients
Verver 2001 Netherlands	Migrants	1993-1998	ACF: entry and every 6 months for 2 years CXR screening programme. Sputum for smear and culture if abnormal CXR.	Smear or culture + Stay <30 months	454	368	Smear positivity of culture confirmed TB patients Symptom duration at diagnosis Treatment outcomes
Churchyard 2000 Free State, South Africa	Miners in 1 company	1993-1997	ACF: annual miniature CXR screening programme. Standard CXR and sputum for smear and culture if abnormal.	Culture + Known HIV status and treatment outcome	1225	1011	Treatment outcomes
Capewell 1986 Edinburgh, UK	Hostel dwellers	1976-1982	ACF: x2/year miniature CXR screening programme, with monetary incentive. Referred to clinic if abnormal CXR.	Culture +	42	26	Smear positivity of culture confirmed TB patients

Clinical review – risk groups cluster randomised trials							
Jenum 2018 Palamaner in Andhra Pradesh, South India	Neonates receiving BCG by 72 hours of birth	2006-2010	ACF: x2/month for 2 years, home visits with screens for symptoms, TB exposure and failure to thrive. Referral with reminders to study medical ward for work up if +.	n/a	2215†	2167†	Mortality – all cause
Fox 2018, 70 districts in 8 provinces of Vietnam	Household contacts in rural and urban areas	2010-2015	ACF: CXR and symptom screen at 0, 6, 12 and 24 months by National TB programme staff at district clinics. Sputum for smear and culture if symptoms or abnormal CXR	n/a	10069†	15638†	Mortality – all cause

Economic review							
Muniyandi 2020 India	General population (rural)	2016-2018	Prevalence survey: house-to house screening with symptoms and CXR. Sputum for smear and culture if symptoms or abnormal CXR.	Adult ≥15 years with TB	110	226	Empirical; CA from patient perspective; Primary costing data; 2018 cost reference year Diagnosis costs - *Direct (medical and non-medical); Indirect* – no input information Treatment costs - *Direct (medical and non-medical); indirect* – no input information
Gurung 2019 Nepal	OPD attendees; social contacts of TB patients; general population (rural);	2018	ACF: Symptom screen in OPD; symptom screen social contacts; general population TB camp with community health worker house-to-house symptom screen 1-2 days before. Sputum for Xpert if symptoms.	Adult ≥15 years with PTB between 2-12 weeks of treatment	50	49	Empirical; CA from patient perspective; Primary costing data; 2018 cost reference year Pre-treatment costs:*Direct medical* – consultation, x-ray, lab tests, drugs, other; *Direct non-medical* – transport, food; *Indirect* – time loss, income loss intensive phase treatment costs:*Direct medical* – consultation, x-ray, drugs; *Direct non-medical* – transport, food; *Indirect* – time loss, income loss
Hussain 2019 Pakistan	Private clinic attendees; general population (urban)	2011-2012	ACF: HCW incentives; symptom screen clinic attendees; ECF: TB IEC to general population. Sputum for smear/Xpert and CXR if symptoms.	TB patients on treatment for at least 2 months	84	45	Decision modelling; CEA from provider and patient perspective; Primary and secondary costing data; 2012 cost reference year Pre-diagnosis costs: *Direct medical* – consultation, tests, drugs; *Direct non-medical* – food and transport Diagnosis costs:*Direct medical* – consultation, tests, drugs; *Direct non-medical* – food and transport Treatment costs:*Direct medical* – consultation, tests, drugs*; Direct non-medical* – food and transport Indirect costs – lost earnings
Shewade 2018 India	Marginalised and vulnerable populationsŦ	2016-2017	ACF: one-off community volunteer house-to house symptom screen. Referral for sputum smear if symptoms.	Smear + Adult ≥15 years newly registered for treatment	234	231	Empirical; CA from patient perspective; Primary costing data; 2018 cost reference year Diagnosis costs:*Direct medical* – consultation, drugs, tests; *Direct non-medical* – travel; *Indirect* – wages/income lost
Morishita 2016 Cambodia	Household and neighbourhood contacts of smear + TB patients	2014	ACF: all household and symptomatic neighbourhood contacts invited for CXR screening on a specific date. Sputum for Xpert if abnormal CXR or symptoms.	New PTB with cured or completed treatment outcome	108	100	Empirical; CA from patient perspective; Primary costing data; 2014 cost reference year Pre-treatment costs: *Direct medical* – administration, tests, x-ray, drugs, hospitalisation; *Direct non-medical* – transport, food, guardian, insurance reimbursement; *Indirect* – lost income from health seeking and sick leave Treatment costs*: Direct medical* –hospitalisation; *Direct non-medical* – transport (DOTS, drug pick-up, follow-up visits), supplemental food, guardian/care giver, interest for borrowed money, insurance re-imbursement; *Indirect* – lost income (patient, guardian/care giver), reduced household activity, value lost from sold property
Sekandi 2015 Uganda	General population (urban)	2012	Prevalence survey: house-to-house symptom screen. Sputum collection if symptoms for smear/culture.	Adult ≥15 years on at least 2 weeks of TB treatment	103		Decision modelling; CEA from societal perspective; Primary and secondary costing data; 2013 cost reference year Diagnosis costs: *Direct non-medical* - transportation, food, care giver, child care/hired help; *Indirect* – patient and care giver time lost

⁎ number of people with TB unless otherwise indicated; PCF=passive case-finding; ACF=active case-finding; + = positive; CXR=chest radiograph; ECF=enhanced case finding; IEC=information, education and communication

¶ community-promoters - individuals with previous experience in community outreach activities who are provided training about TB).

Ŧ includes slums, tribal areas, scheduled caste communities, areas where occupational lung diseases is high, areas where individuals with high risk of acquiring TB reside including stone crushing/mining/weaving industry/unorganized labour (construction workers etc)/homeless, high HIV/AIDS burden areas, areas or communities with high TB incidence (including prisons) and among household contacts of sputum smear positive TB patients.

ʃ Papers report different outcomes on the same study participants; BCG=Bacillus Calmette–Guérin; n/a=not applicable.

† total number in screened and passive case-finding group; CA=cost analysis; OPD=outpatient department; PTB=pulmonary TB; x-ray=radiography; HCW=health care worker; CEA=cost effectiveness analysis; DOTS=Directly Observed Treatment, Short-course.

Fifteen were observational studies. The characteristics of TB patients identified through screening and PCF varied across these studies (Table 3, Table 4, Table 5). All had a high risk of bias for the outcomes identified (Appendix 2); most (n=11/15) did not adjust for potential confounders.

**Table 3:** Smear grade 3+ and smear positivity among culture confirmed TB patients reported in n=8 observational studies

First author, country and population, screening tool	Group	Smear grade 3+ / all smear positives		Smear + / culture confirmed		Prevalence ratio (screen/PCF)	Comments
		n/N*	% (95%CI)	n/N⁎⁎	% (95%CI)		
General population							
Abdurrahman 2016 Nigeria Symptoms	Screen	101/480	21% (17-25%)	-	-	0.46	Diagnosed TB patients Screened vs PCF - screened group more likely to be older, married and less likely to be HIV infected.
	PCF	96/208	46% (39-53%)	-	-		
den Boon 2008 South Africa Smear & culture	Screen	6/18	33% (13-59%)	-	-	0.63	Denominator for smear grade - screened group includes those lost to follow-up pre-treatment; PCF those starting treatment only Diagnosed in screened and on treatment in PCF groups - no difference in age and gender.
	PCF	234/446	52% (48-57%)	-	-		
Santha 2003 India CXR and symptoms	Screen	3/96	3% (1-9%)	-	-	0.07	Denominator for smear grade - screened group includes those lost to follow-up pre-treatment; PCF those starting treatment only All (smear +ve and -ve) diagnosed in screened and on treatment in PCF groups - screened group more likely to be older, male, illiterate, sole earner, have poor quality house and a 1 room house
	PCF	139/330	42% (37-48%)	-	-		
Risk groups							
Shewade 2019 India: Marginalised/vulnerable† Symptoms	Screen	39/233	17% (12-22%)	-	-	0.84	On treatment TB patients Screened vs PCF- screened group more likely to be older, from rural areas and live further from microscopy units.
	PCF	53/265	20% (15-25%)	-	-		
Paiao 2016 Brazil: Prisoners Symptoms	Screen	-	-	4/40	10% (3-24%)	0.20	Diagnosed TB patients
	PCF	-	-	27/53	51% (37-65%)		
Story 2012 UK: Homeless people, drug users, prisoners, asylum seekers CXR	Screen	-	-	11/23	48% (27-69%)	0.67	On treatment TB patients Association between screening and smear positivity maintained after adjusting for age and gender
	PCF	-	-	104/146	71% (63-78%)		
Verver 2001 Netherlands: Migrants CXR	Screen	-	-	60/159	38% (30-46%)	0.68	On treatment TB patients Screened vs PCF - screen detection varied by country of origin, decreased with increasing length of stay and was less likely among illegal migrants.
	PCF	-	-	59/107	55% (45-65%)		
Capewell 1986 UK: Hostel dwellers CXR	Screen			11/16	69% (41-89%)	0.87	On treatment TB patients
	PCF			15/19	79% (54-94%)		

⁎ n/N=number with smear grade 3+/total number with smear grade scanty, 1+, 2+ and 3+.

⁎⁎ n/N=number smear positive/total number culture positive; 95%CI = 95% confidence interval; PCF=passive case-finding.

† included slums, tribal areas, scheduled caste communities, areas where occupational lung diseases is high, areas where individuals with high risk of acquiring TB reside including stone crushing/mining/weaving industry/unorganized labour (construction workers etc)/homeless, high HIV/AIDS burden areas, areas or communities with high TB incidence (including prisons) and among household contacts of sputum smear positive TB patients; CXR=chest radiograph.

### 3.2 General populations

Eight observational studies were conducted in rural and/or urban populations; all were from South Asia and sub-Saharan Africa [[20], [21], [22], [23], [24], [25], [26], [27]]. Most (n=7/8) involved one-off house-to-house ACF strategies (n=5/7 were prevalence surveys) [[20], [21], [22], [23], [24], [25],^27^]. Four (50%) used symptom screening, [20,22,26,27] three (38%) chest radiographs (CXRs) and symptoms, [[23], [24], [25]] and one (12%) prevalence survey conducted sputum smear and culture on all individuals [21].

Three studies [20,21,25] reported on smear grade (Table 3 showing proportions and prevalence ratios and Appendix 3). All showed screened TB patients were less likely to have higher smear grades, but the small sample size of the screened group gave wide CIs in one [21]. Two studies conducted in the same south Indian population over consecutive calendar periods reported on pre-treatment LTFU (Table 4) [23,24]. In both, the proportion LTFU among those screened was higher (screened 23% and 29% versus PCF 15% and 14%). Among individuals LTFU, none died in the screened group, while nearly 20% had died in the PCF group for whom outcomes were available [23]. Symptom duration was longer in the PCF group in one study (cough <3 weeks 13% in PCF versus 28% in screened group) [25] but shorter in another (mean cough duration 6.8 weeks in PCF versus 10.3 weeks in screened group) [20]. One study found no difference in time to treatment start between screened and PCF groups [22].

**Table 4:** Pre-treatment LTFU, time from symptoms to first contact with health services, diagnosis and treatment start reported in n=7 observational studies

First author, Population	Screening tools TB case definition	Outcomes						Comments
General population								
Pre-treatment LTFU				N	n	%	95%CI	
Gopi 2005 India	CXR and symptoms Smear +ve	-	Screened	243	57	23	18-29	Screened group – no deaths. Reasons for defaulting included not interested in initiating treatment, symptoms too mild, too sick/old and work-related problems. PCF group – 19% died from among those for whom a default reason was known.
			PCF	1049	156	15	13-17	
Balasubramanian 2004 India	CXR and symptoms Smear +ve	-	Screened	231	68	29	24-36	
			PCF	833	120	14	12-17	


Time to first contact with health services				N	n	%	p-value	


Santha, 2003 India	CXR and symptoms Smear +ve	Cough <3 weeks	Screened	96	27	28	<0.001	Baseline characteristics of all (smear +ve and -ve) diagnosed in screened and on treatment in PCF groups - screened group more likely to be older, male, illiterate, sole earner, have poor quality house, 1 room house, lower smear grade and new smear -ve disease.
			PCF	272	35	13		


Time to diagnosis				N	Mean	SD	p-value	

Abdurrahman 2016^∆^ Nigeria	Symptoms Smear +ve	Cough duration in weeks	Screened	485	10.3	2.4	<0.001	Baseline characteristics of diagnosed TB patients (screened vs PCF) - screened group more likely to be older, married and less likely to be HIV infected.
			PCF	209	6.8	2.6		


Time to treatment				N	n	%	p-value	

Shargie, 2006 Ethiopia	Symptoms or on TB treatment Smear +ve	Symptom ≤90 days	Screened	13	6	46	1	Baseline characteristics of on treatment TB patients (screened vs PCF) - screened group younger and a higher proportion were women.
			PCF	24	10	42		


Risk groups								

Time to diagnosis				N	Median	IQR	p-value	

Shewade, 2019 India Marginalised/ vulnerable populations*	Symptoms Smear +ve	Patient-level diagnosis delay from sputum eligible^†^ (days)	Screened	225	12	3-31	0.999	Baseline characteristics of on treatment TB patients (screened vs PCF)- screened group more likely to be older, from rural areas, less educated and live further from microscopy units. Adjusted analysis showed no association between patient-level delay and case-finding, but showed reduction in total diagnosis delay among those screened (screened versus PCF linear regression of log transformed delay in days after adjusting for confounders and clustering beta coefficient -0.31; 95%CI -0.62 to 0.00; p=0.052; screened versus PCF adjusted prevalence ratio for delay ≥50 days 0.77; 95%CI 0.63-0.94; p=0.009)
			PCF	230	10	3-43		
		Health system diagnosis delayŦ (days)	Screened	229	5	0-61	0.008	
			PCF	229	19	1-76		
		Total diagnosis delay^¶^ (days)	Screened	229	45	18-106	0.131	
			PCF	230	61	20-121		
Verver, 2001 Netherlands Migrants	CXR Smear or culture +ve	Symptom duration in weeks among those reporting symptoms	Screened	142	0.0	-	<0.001^ʃ^	Baseline characteristics of on treatment TB patients (screened vs PCF) - screen detection varied by country of origin, decreased with increasing length of stay and was less likely among illegal migrants.
			PCF	332	7.5	-		


Time to treatment				N	Median	IQR	p-value	

Shewade, 2019 India Marginalised/ vulnerable populations*	Symptoms Smear +ve	Total treatment delay from sputum eligible^ï^ (days)	Screened	227	52	22-112	0.37	Baseline characteristics of on treatment TB patients (screened vs PCF)- screened group more likely to be older, from rural areas, less educated and live further from microscopy units. Adjusted analysis showed no association with case-finding (screened versus PCF linear regression of log transformed delay in days after adjusting for confounders and clustering beta coefficient -0.20; 95%CI -0.50 to 0.10; p=0.181).
			PCF	229	62	23-128		

LTFU=loss to follow-up; pre-treatment LTFU=default between diagnosis and treatment start; N=total number of people with TB; n=number with outcomes; %=proportion; 95%CI=95% confidence interval; CXR=chest radiograph; +ve=positive; PCF=passive case-finding; -ve=negative; IQR=interquartile range; SD=standard deviation; ∆Other symptom (fever, weight loss, chest pain and anorexia) durations to diagnosis were assessed, only weight loss was significantly higher in the screened population compared to passively found TB patients;*included slums, tribal areas, scheduled caste communities, areas where occupational lung diseases is high, areas where individuals with high risk of acquiring TB reside including stone crushing/mining/weaving industry/unorganized labour (construction workers etc)/homeless, high HIV/AIDS burden areas, areas or communities with high TB incidence (including prisons) and among household contacts of sputum smear positive TB patients; †patient diagnosis delay=from sputum eligible (15^th^ day of continuous cough/fever or day of the first episode of haemoptysis) to first visit to health care provider.

Ŧ health system diagnosis delay=from first visit to health care provider to date of diagnosis; ¶total diagnosis delay=from eligible for sputum examination to diagnosis*;* ʃsimilar difference observed when results were restricted to n=99 with smear positive disease; ïtotal treatment delay= from sputum eligible (15^th^ day of continuous cough/fever or day of the first episode of haemoptysis) to treatment start.

Four studies involving different screening strategies (symptom; CXR; and smear/culture screening) reported on treatment outcomes (Table 5 showing proportions and prevalence ratios). In three the proportions with treatment success among screened and PCF groups was similar, ranging from 68-80% [21,25,26]. Two studies also reported on pre-treatment LTFU; both only provided data for the screened group (26-32%) [21,25]. There was no difference in the proportion who died between screened (range 6-8%) and PCF (range 4-11%) groups in four studies [21,[25], [26], [27]]. There was no difference in the proportion LTFU during TB treatment between screened (range 6-20%) and PCF (range 8-19%) groups [25,26].

**Table 5:** On-treatment outcomes (treatment success, case fatality and default on-treatment) among smear, Xpert and/or culture positive TB patients reported in n=7 observational studies and n=1 CRT, and, all-cause mortality reported in n=2 CRT

Observational studies												
First author, country and population, screening tool	Group	Treatment success		PR	Case fatality		PR	LTFU on treatment		Pre-treatment LTFU*		Comments
		n/N	% (95%CI)		n/N	% (95%CI)		n/N	% (95%CI)	n/Nʃ	(%)	
General population												
den Boon 2008 South Africa smear & culture	Screen	16/20	80% (56-94%)	1.00	2/27	7% (1-24%)	1.95	-	-	7/27	26%	Denominator for case fatality - screened group includes those LTFU pre-treatment; PCF those starting treatment only. Baseline characteristics of diagnosed in screened and on treatment in PCF groups - no difference in age, gender, smear grade between groups.
	PCF	379/473	80% (76-84%)		18/473	4% (2-6%)		-	-	-	-	
Santha 2003 India CXR and symptoms	Screen	45/65	69% (57-80%)	1.01	4/65	6% (2-15%)	0.88	13/65	20% (11-32%)	31/96	32%	Baseline characteristics of all (smear +ve and -ve) diagnosed in screened and on treatment in PCF groups - screened group more likely to be older, male, illiterate, sole earner, have poor quality house, 1 room house, lower smear grade and new smear -ve disease.
	PCF	225/330	68% (63-73%)		23/330	7% (4-10%)		63/330	19% (15-24%)	-	-	
Harper 1996 Nepal Symptoms	Screen	50/64	78% (66-87%)	1.00	5/64	8% (3-17%)	0.96	4/64	6% (2-15%)	-	-	Baseline characteristics of diagnosed TB patients (screened vs PCF) – screened more likely to be female (and age among women tended to be older).
	PCF	997/1272	78% (76-81%)		104/1272	8% (7-10%)		96/1272	8% (6-9%)	-	-	
Cassel 1982 Nepal Symptoms	Screen	-	-		9/111	8% (4-15%)	0.76	-	-	11/111	10%	Denominator for case fatality - screened group includes those LTFU pre-treatment; PCF group are those starting treatment. Baseline characteristics of diagnosed TB patients (screened vs PCF) – screened group were older and the male to female ratio was lower.
	PCF	-	-		17/159	11% (6-17%)		-	-	-	-	

Risk groups												
Shewade 2019 India; Marginalised and vulnerable† Symptoms	Screen	247/274	90% (86-93%)	1.03	7/274	3% (1-5%)	0.69	16/274	6% (3-9%)	-	-	Baseline characteristics of on treatment TB patients (screened vs PCF)- screened group more likely to be older, from rural areas and live further from microscopy units. No association between screening and treatment success after adjusting for age, gender and distance from microscopy unit.
	PCF	260/296	88% (83-91%)		11/296	4% (2-7%)		22/296	7% (5-11%)	-	-	
Verver 2001 Netherlands: Migrants CXR	Screen	384/454	85% (81-88%)	1.06	1/454	0.2% (0-1%)	0.07	47/454	10% (8-14%)	-	-	Baseline characteristics of on treatment TB patients (screened vs PCF) - screen detection varied by country of origin, decreased with increasing length of stay and was less likely among illegal migrants.
	PCF	293/368	80% (75-84%)		12/368	3% (2-6%)		36/368	10% (7-13%)	-	-	
Churchyard 2000 South Africa: Miners CXR	Screen	-	-		12/1225	1% (0.5-2%)	0.14	-	-	-	-	Baseline characteristics of on treatment TB patients (screened vs PCF) - screened less likely to be HIV infected. After adjusting for HIV status, sputum status, treatment category, age, disease extent on CXR, silicosis and drug resistance, association between PCF and case fatality maintained (PCF versus screened aOR 5.6; 95%CI 2.6-12.2)
	PCF	-	-		69/1011	7% (5-9%)		-	-	-	-	

Cluster randomised controlled trials												

First author, country and population, screening tool	Community, number and baseline data								Results			

General population												
Shargie 2006^∆^ Ethiopia: Symptoms	87 contiguous administrative units clustered into 32 communities 32 communities randomised – 12 to screening and 20 to PCF N^Ŧ^ smear +ve TB patients - screen=159; PCF=221 Follow-up during treatment Communities and TB patients - similar baseline characteristics between groups								Treatment success: screen vs PCF		n=128 (81%) vs n=165 (75%); difference (95%CI) 6 (-4 to 15); p=0.12	
									Death: screen vs PCF		n=5 (3.1%) vs n=7 (3.2%); difference (95%CI) -0.1 (-4 to 4); p=0.49	
									LTFU on treatment: screen vs PCF		n=26 (16%) vs n=48 (22%); difference (95%CI) -6 (-14 to 3); p=0.11	

Risk groups												
Jenum 2018 India: neonates Symptoms	Cluster – villages or subsection of towns 592 clusters randomised (8 strata) – 297 to screening and 295 to PCF N^Ŧ^ in each group - screen=2215; PCF=2167 Follow-up 2 years Study groups – PCF group had more Hindus, lower paternal literacy and higher use of wood/agricultural residues for fuel. No difference in other characteristics								All-cause mortality: screen vs PCF		n=49 (2.2%) vs n=71 (3.3%); aOR¶ (95%CI) 0.68 (0.47-0.98)	
									Cause of death: screen vs PCF		Reduction in deaths due to pneumonia/respiratory infections (aORƳ 0.34; 95%CI 0.14-0.80).	
									LTFU: screen vs PCF		n=38 (1.7%) vs n=60 (2.8%); aOR¶ (95%CI) 0.62 (0.41-0.94)	
Fox 2018 Vietnam: household contacts CXR and symptoms	70 of 112 districts in 8 Vietnamese provinces selected with probability proportional to population. 70 districts randomised – 36 to screened and 34 to PCF N^Ŧ^ in each group - screen=10,069; PCF=15,638 Follow-up 2 years Study groups – PCF group household size higher and lower proportion reported prior history of TB.								All-cause mortality: screen vs PCF		n=60 (0.6%) vs 265 (1.7%); RR (95%CI) 0.60 (0.50-0.80)	

CRT=cluster randomised controlled trial; PR=prevalence ratio (screened/passive case finding population); LTFU=loss to follow-up.

⁎ pre-treatment LTFU =lost to follow-up between diagnosis and treatment start; n/N=number with outcome/total number *started on TB treatment* (unless otherwise indicated); 95%CI = 95% confidence interval.

ʃ n/N=number lost to follow-up pre-treatment/total number diagnosed with TB.

† included slums, tribal areas, scheduled caste communities, areas where occupational lung diseases is high, areas where individuals with high risk of acquiring TB reside including stone crushing/mining/weaving industry/unorganized labour (construction workers etc)/homeless, high HIV/AIDS burden areas, areas or communities with high TB incidence (including prisons) and among household contacts of sputum smear positive TB patients; PCF=passive case-finding; CXR=chest radiographs; +ve=positive; -ve=negative; aOR=adjusted odds ratio; ŦDenominator in each study group.

¶ adjusted for clustering, gender, religion, father's education and fuel type used.

Ƴ adjusted for clustering, gender, religion and father's education; RR=relative risk; ∆Data not shown in table - weighted mean of median pre-treatment symptom duration 89 days in screened vs 136 days in control group (difference [95%CI] -47 [-76 to -19]; p=0.001).

One CRT, conducted in 32 contiguous rural Ethiopian communities with difficult access to health care, used monthly ECF with outreach clinics to initiate diagnosis (continued at health facilities through routine services) over 1 year in 12 intervention communities (Table 2, Table 5 and Appendix 2) [28]. There was no difference in TB patient characteristics, treatment success, on-treatment case fatality or on-treatment LTFU by study arm. Data on pre-treatment LTFU was not provided. But pre-treatment symptom duration was significantly lower in the intervention group (median difference between intervention and control group -47 days; 95%CI -76 to -19; 55-60% reduction in duration in the last three quarters compared to the first quarter in intervention communities, with corresponding 3-20% fall in control communities). Because of insufficient information to assess one bias domain, the risk of bias assessment raised some concerns.

### 3.3 Risk groups

Seven observational studies reported on risk groups, including prisoners,[[29], [30], [31], [32]] migrants, [33] miners, [34] and homeless people. [32,35]. Four involved established European and South African CXR screening programmes [[32], [33], [34], [35]]. Three studies from India and Brazil reported on one-off/limited ACF using symptoms [[29], [30], [31]].

One Indian study found no difference in smear grade among screened and PCF groups (Table 3 showing proportions and prevalence ratios) [29]. Three European and one Brazilian study reported on smear positivity among culture-confirmed TB patients [[31], [32], [33],^35^]. The proportion with positive smears was lower in those screened in three [[31], [32], [33]]. One study showed no association but small sample sizes gave wide CIs in both study groups [35]. No studies reported on pre-treatment LTFU (Table 4). Symptom duration was shorter in the screened group in two studies (prevalence of diagnosis delay ≥50 days was 23% lower in the screened group in an Indian study, [30] and the median symptom duration was 7.5 weeks in the PCF versus 0.0 weeks in the screened group in a study from the Netherlands [33]. Time to treatment start in one Indian study [30] found no difference between the screened and PCF groups.

Three studies (including two established CXR screening programmes) reported on treatment outcomes (Table 5 showing proportions and prevalence ratios). The proportions with treatment success among screened and PCF groups was similar, ranging from 80-90% in two [29,33]. In one Indian study reporting on one-off symptom screening, there was no difference in case fatality among screened and PCF groups [29]. Two studies reporting on ∼4-5 years of data from established CXR screening programmes among migrants to the Netherlands and South African miners showed higher case fatality among the PCF group (PCF versus screened odds ratio [OR] 15.3; 95%CI 2.0-118.0; adjusted OR 5.6; 95%CI 2.6-12.2 respectively) [33,34]. There was no difference in the proportion LTFU during TB treatment between screened (range 6-10%) and PCF (range 7-10%) groups [29,33].

Two CRTs were identified (Table 2, Table 5 and Appendix 2) [36,37]. One among Indian neonates compared fortnightly ACF over 2 years, in 297 intervention communities to PCF in 295 control communities [36]. Screening was associated with lower all-cause mortality compared to PCF (adjusted OR 0.68 [95%CI 0.47-0.98]), which was attributed to decreases in pneumonia/respiratory infections. The risk of bias was high which could work to underestimate the effect of screening on mortality. A CRT among Vietnamese household contacts of TB patients, compared CXR and symptom screening at 0, 6, 12 and 24 months in 36 intervention communities to PCF in 34 control communities [37]. Screening was associated with lower all-cause mortality compared to PCF (risk ratio 0.60 [95%CI 0.50-0.80]). The risk of bias assessment raised some concerns as the data represented a post-hoc analysis.

### 3.4 Economic review

**Figure 2:**
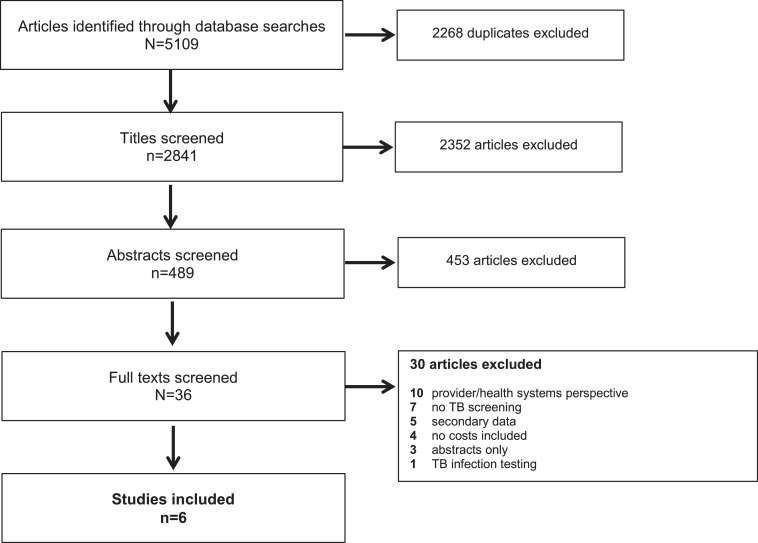
Study selection process - flow diagram of number of original research articles considered for the economic review

From 2841 articles, six observational studies were eligible [[38], [39], [40], [41], [42], [43]] (Figure 2 and Table 2); none were included in the previous review. Most were from South Asia (n=4; 67%), [[38], [39], [40], [41]] with one from South East Asia, [42] and one from sub-Saharan Africa [43]. Most studies included general populations (n=4; 67%); [[38], [39], [40],^43^] three involved house-to-house screening [38,39,43]. Risk groups were those with structural risk factors (n=1), [41] household and neighbourhood contacts (n=1), [42] and social contacts (n=1) [39] of TB patients, and health facility attendees (n=2) [39,40]. Four studies (67%) used symptom screening alone, [[39], [40], [41],^43^] whereas two (33%) used CXR and symptoms. [38,42]. The analyses undertaken varied; four performed cost analysis [38,39,41,42] and two conducted cost-effectiveness analysis [40,43]. All studies reported findings transparently; three [[38], [39], [40]] met all CHEERS checklist criteria (Appendix 4).

Data were summarised using different measures (means, medians). The illness periods for which costs were reported varied; two studies reported diagnosis costs alone, [41,43] two pre-treatment and treatment costs, [39,42] one diagnosis and treatment costs, [38] and one pre-diagnosis, diagnosis and treatment costs [40] (Table 2 and 6; Appendix 5). While cost inputs and granularity of reporting varied across studies, all calculated aggregated costs for the reported illness period (Table 6 and Appendix 5). In all studies, higher total costs were incurred in the PCF compared to screened group. Four studies assessed catastrophic cost prevalence, which was higher in the PCF (range 12-61%) compared to screened (range 9-45%) group [38,39,41,42]. In two Indian studies, using house-to-house screening among general populations [38] and those with structural risk factors, [41] total costs and catastrophic costs (on multivariable analysis) were significantly lower in the screened compared to PCF groups. In two studies with small sample sizes, among Cambodian household and neighbourhood contacts of TB patients [42] and among mainly outpatient attendees and social contacts of TB patients in Nepal, [39] there was no statistically significant difference in total costs and catastrophic costs on univariable analysis between screened and PCF groups. Two studies did not assess differences in mean total costs or report catastrophic costs [40,43].

**Table 6:** Costs for the entirety of the illness period and the prevalence of catastrophic costs from n=6 studies reporting on patient costs*

First author, population and screening method, illness period and costs reported	Combined cost for the illness period (US$)			Catastrophic cost prevalence				Comments
	Screen	PCF	p-value	Screen	PCF	p-value		
Muniyandi (2020); India General population; symptoms and CXR screen Diagnosis and treatment Direct (medical and non-medical) and indirect costs	Mean (SEM)	69 (18)	227 (20)	0.001	9%	29%	-	Screened group more likely to be older, illiterate, smoke and report no symptoms. No data on bacteriological status. On adjusted analysis catastrophic costs were significantly higher among the PCF group (aOR 3.68; 95%CI 1.62-8.33)
Gurung (2019); Nepal OPD attendees, social contacts of people with TB, general population TB camps; symptom screen Pre-treatment (from symptom start) and intensive treatment phase Direct (medical and non-medical) and indirect costs	Median (IQR)	253 (81–453)	315 (126–544)	0.16	45%	61%	0.14	60% OPD; 34% social contacts; 6% camps No difference in socio-demographic, disease and health seeking characteristics between groups. PCF group interviewed >1 month after treatment start (∼70%) reported lower costs than those interviewed within 1 month. No difference seen with screened group.
Shewade (2018); India Marginalised/vulnerable populations⁎⁎; symptom screen From sputum eligible¶ to diagnosis Direct (medical and non-medical) and indirect costs	Median (IQR)	5 (0-40)	20 (4-69)	<0.001	10%	12%	-	Screened group more likely to be older, from rural residence, have no formal education, have lower median monthly income and not report weight loss. No significant difference in smear grade, weight in Kg, haemoptysis or fever between screened and PCF group On adjusted analysis catastrophic costs were significantly lower among the screened group (aPR 0.68; 95%CI 0.69-0.97)
Morishita (2016); Cambodia HH and neighbourhood contacts; CXR screen Pre-treatment and during 6 months of treatment Direct (medical and non-medical) and indirect costs	Median (IQR)	241 (66–595)	290 (114–813)	0.10	36%	45%	0.24	No difference in socio-demographic characteristics. PCF group more likely to be smear/Xpert positive and live near health centres. No other clinical data provided
Hussain (2019); Pakistan HCW - incentives; clinic attendees – symptom screen; general population – TB IEC Pre-diagnosis, diagnosis and treatment phase Direct (medical, non-medical) and indirect costs	Mean†	59	71	NR	NR			52% smear negative in screened group and 42% smear negative in PCF group
Sekandi (2015); Uganda General population; symptom screen Diagnosis Direct (non-medical) and indirect costs	Mean (range)	5 (2–7)	29 (14–43)	NR	NR			

⁎ All values (costs and proportions) rounded to the nearest whole number; PCF=passive case-finding; CXR=chest radiograph; SEM=standard error of the mean; aOR=adjusted odds ratio; 95%CI=95% confidence interval; OPD=outpatient department; IQR=interquartile range

⁎⁎ included slums, tribal areas, scheduled caste communities, areas where occupational lung diseases is high, areas where individuals with high risk of acquiring TB reside including stone crushing/mining/weaving industry/unorganized labour (construction workers etc)/homeless, high HIV/AIDS burden areas, areas or communities with high TB incidence (including prisons) and among household contacts of sputum smear positive TB patients

¶ from 15^th^ day of continuous cough, fever or the day of the 1^st^ episode of haemoptysis; aPR=adjusted prevalence ratio; HH=household; HCWs=health care workers; IEC=information, education and communication

† no measure of spread reported; NR=not reported

## 4 Discussion

We synthesised literature published between 1980-2020, to generate up-to-date evidence for the individual effects of TB screening. We found very few studies addressing the review questions. The WHO END-TB strategy sets out ambitious targets to reduce TB death, incidence and catastrophic costs by 2035 [44]. At the 2018 United Nations General Assembly high-level meeting, world leaders reaffirmed their commitment to ending TB [45,46]. At a time of unprecedented political commitment to find, test and treat TB patients, evidence for strategies such as TB screening to inform in-country decision making globally, is vital. Further, the reversal in TB control efforts and case-detection due to the COVID-19 pandemic [47,48] may going forward, make TB screening even more important.

A general challenge with interpreting the findings is the observational design of most studies. This is compounded by differences in reported outcome measures, insufficient data on the care cascade, unadjusted analyses, small sample sizes, and length-time bias (where screening may detect individuals with less severe indolent disease who may have different characteristics, longer disease course and better outcomes including survival, than those who are identified through PCF). These limitations must be kept in mind when interpreting results. Definitive evidence for the effects of TB screening requires well-conducted RCTs. However, these require large sample sizes, long term follow-up and are resource intensive. We only identified three RCTs, conducted over relatively short time-periods (1-2 years) [28,36,37]. Therefore, insights from routine programme implementation are essential. While overall screening approaches will depend on the context and available resources, general principles dictate that screening is not one-off, is integrated into health systems, with quality-assured diagnosis and treatment services [4,7]. We only identified four studies (all in risk groups) reporting on established screening programmes [[32], [33], [34], [35]]. But there was general consistency in most findings, irrespective of the screening strategy used.

TB screening, by engaging individuals earlier into care, should result in earlier diagnosis when disease is less severe [4]. Smear grade and proportion smear positive among culture-confirmed TB patients was lower in the screened group in most studies with larger sample sizes, suggesting screening does identify individuals with less severe disease. Length-time bias may explain this. But the reported reduction in pre-diagnosis symptom duration among those screened, while subject to recall bias, suggests earlier diagnosis plays a role. If individuals are identified earlier, when disease is less severe, and linked to care, this should translate to better outcomes for the individual [4].

Studies consistently showed no difference in treatment success between screened and PCF groups. This could be a true finding (screening does not improve treatment success). Or it may be due to potential confounders or the inherent limitations of routine data, where identifying TB patients screened from those self-presenting can be challenging and successful outcomes may be over-ascertained, potentially biasing the effect towards the null. Data on pre-treatment LTFU, while limited and not generalisable, suggests pre-treatment LTFU is high among screened TB patients; in one study, no deaths were reported in the screened group [23]. In the PCF group, there was high pre-treatment case fatality, [23] similar to other reports [49]. Therefore, on-treatment outcomes, which ignore deaths pre-treatment, may underestimate the effects of screening.

Two studies (Churchyard 2000 and Verver 2001) found screening was associated with lower case fatality, [33,34] but due to their observational nature we cannot exclude length-time bias and uncontrolled confounders. Both report on established CXR screening programmes, with large sample sizes, access to good health systems and better reporting of deaths. While neither study report on pre-treatment LTFU, individuals treated could be more representative of those diagnosed. Churchyard 2000, among miners did not report treatment success by screened and PCF groups [34]. Verver 2001, showed no difference in treatment success, [33] but this study among migrants, had few deaths overall which may reflect a healthy migrant effect, giving better overall outcomes across study groups. Two CRTs (Jenum 2018 in neonates and Fox 2018 in household contacts of TB patients) found screening was associated with lower all-cause mortality, [36,37] with Fox 2018, showing no difference in on-treatment outcomes (among all TB patients) between study groups [37]. The limitations of these CRTs (generalisability, post-hoc analysis) need to be borne in mind when interpreting findings. But, in line with these are RCTs comparing different screening strategies in risk groups, showing lower mortality/case fatality among individuals, especially with severe disease, receiving more intensive screening [50,51]. As all data represent risk groups, findings cannot be extrapolated to general populations.

Pre-treatment LTFU, while likely to be setting-specific, can be frequent with interventions targeting “well” individuals. Programmes should ensure that all individuals diagnosed are linked to treatment, with context-specific barriers to engaging with care identified and mitigated. A CRT in rural Ethiopia where health care access is difficult, compared ECF to ECF plus community-based care (sputum collection, providing treatment and supporting adherence) by community health workers over one year [52]. Treatment success was significantly higher in the latter group, highlighting how combining screening with strategies that minimise pre-treatment LTFU can increase treatment success. Further, if all individuals diagnosed at an earlier stage are not started on treatment, reducing transmission, population-level benefits [4] shown in trials [53,54] may not be realised.

Due to the limitations of the identified economic studies (e.g. differences in the cost inputs and illness periods; small sample sizes; recall bias; and unadjusted analyses) we cannot directly compare findings between studies. Further, the data are mostly from South Asia, limiting generalisability. Nevertheless, all studies consistently showed lower total costs and catastrophic cost prevalence among those screened. While we did not assess screening costs/cost-effectiveness from a health system perspective, this can be high. When viewed from a societal perspective, there may be potential offsets to these costs. But, given the limitations of the included studies, only cautious conclusions can be drawn. Patient costs are often reported as barriers to accessing TB care.^8^,[55], [56], [57] Therefore, standardising the collection and reporting of patient cost inputs as part of routine programme monitoring could help identify how interventions affect this patient important outcome, guiding policy making.

These reviews have several limitations. We only searched four databases; the grey literature was not searched. Only English, French and Spanish articles were included. The economic review only included articles from 2010. Therefore, some relevant articles may have been missed. As studies were heterogeneous, we could not meta-analyse the data. We did not assess publication bias.

An important finding was the limited data on individual outcomes, despite many publications on TB screening studies/programmes [58]. Going forward, studies/programmes must prioritise reporting this data, along with the screening cascade. Evaluations should be carefully designed, to identify appropriate control groups and adjust for potential confounders, allowing valid comparisons across diagnosed TB patients in screened and unscreened populations.

In conclusion, we found very limited data on the effect of TB screening on individual outcomes. Routine/research programmes must prioritise collecting and reporting this data.

## Data sharing statement

All data are included within the article and supplementary material.

## Declaration of Competing Interest

LT reports WHO consultancy work for the guideline development process and a Clinical Research Training Fellowship from the MRC (Grant Ref: MR/N020618/1).

LHC reports a contract from WHO TB Programme to Jonathan Golub for systematic review of ACF for TB and sub-contract/consulting for JHU for systematic review of ACF for TB.

JEG received a contract provided to Johns Hopkins University to conduct systematic reviews for the WHO's TB screening guidelines; received an NIH grant to conduct TB case finding in India, a second to test for and treat latent TB infection in Brazil; received UNITAID grants to conduct implementation research around latent TB infection in several African countries; and sat on the Scientific Advisory Board for the Aurum Institute in November 2019.

CM is a salaried staff of the WHO and is involved in policy development on TB. CM alone is responsible for the views expressed in this publication and they do not necessarily represent the decisions or policies of WHO.

ELC has received a Wellcome Trust Senior Research Fellowship in Clinical Science: 200901/Z/16/Z to their institution.

RMB reports salary support from my Wellcome Trust Clinical PhD fellowship, awarded through her institution, grant number 203905/Z/16/Z; received payment from WHO to her institution for work on systematic review linked to this present review (but different to this review).

PM reports that he is funded by Wellcome (206575/Z/17/Z).

EK has a consultancy contract with LSHTM for other work, this work was done under that umbrella.

HMA reports WHO consultancy for the work for the guideline development process; reports that EDCTP fund the larger TREATS consortium as a grant paid to her institution that covers some of her time; reports that she is a member of the technical review panel of the Global Fund and receive honoraria for her work.

All other authors have nothing to declare.

The designations used and the presentation of the material in this publication do not imply the expression of any opinion whatsoever on the part of WHO concerning the legal status of any country, territory, city or area, or of its authorities, nor concerning the delimitation of its frontiers or boundaries.

## Appendix Supplementary materials

Image, application 1

## References

[1] World Health Organization (2021). https://apps.who.int/iris/bitstream/handle/10665/336069/9789240013131-eng.pdf.

[2] Harries AD, Lin Y, Kumar AMV, Satyanarayana S, Takarinda KC, Dlodlo RA (2018). What can National TB Control Programmes in low- and middle-income countries do to end tuberculosis by 2030?. F1000Res.

[3] Ho J, Fox GJ, Marais BJ. (2016). Passive case finding for tuberculosis is not enough. Int J Mycobacteriol.

[4] World Health Organization. Systematic screening for active tuberculosis: principles and recommendations 2013 [Available from: https://apps.who.int/iris/bitstream/handle/10665/84971/9789241548601_eng.pdf;jsessionid=DB3EB91C825BD64C12255EAB7EFAC190?sequence=1. Accessed 1 April 2020]. 25996015

[5] Lonnroth K, Corbett E, Golub J, Godfrey-Faussett P, Uplekar M, Weil D (2013). Systematic screening for active tuberculosis: rationale, definitions and key considerations. Int J Tuberc Lung Dis.

[6] Tiemersma EW, van der Werf MJ, Borgdorff MW, Williams BG, Nagelkerke NJ. (2011). Natural history of tuberculosis: duration and fatality of untreated pulmonary tuberculosis in HIV negative patients: a systematic review. PloS one.

[7] World Health Organization (1968).

[8] Tanimura T, Jaramillo E, Weil D, Raviglione M, Lonnroth K. (2014). Financial burden for tuberculosis patients in low- and middle-income countries: a systematic review. Eur Respir J.

[9] Mood C, Jonsson JO. (2016). The Social Consequences of Poverty: An Empirical Test on Longitudinal Data. Soc Indic Res.

[10] Marmot M. (2002). The influence of income on health: views of an epidemiologist. Health Aff (Millwood).

[11] Kranzer K, Afnan-Holmes H, Tomlin K, Golub JE, Shapiro AE, Schaap A (2013). The benefits to communities and individuals of screening for active tuberculosis disease: a systematic review. Int J Tuberc Lung Dis.

[12] Higgins JPT, Thomas J, Chandler J, Cumpston M, Li T, Page MJ (2021). Cochrane Handbook for Systematic Reviews of Interventions version 6.2. Cochrane.

[13] Schünemann H, Brożek J, Guyatt G, Oxman A, editors. GRADE handbook for grading quality of evidence and strength of recommendations Updated October 2013 [Available from: https://gdt.gradepro.org/app/handbook/handbook.html. Accessed 1 June 2020].

[14] World Health Organization (2013).

[15] Chaisson L. (2020). Overview and systematic review of the number needed to screen for active TB. 51st World Conference on Lung Health of the International Union Against Tuberculosis and Lung Disease (The Union); 21st October. The International Journal of Tuberculosis and Lung Disease.

[16] Global Health Cost Consortium. The Unit Cost Study Repository [Available from: https://ghcosting.org/pages/data/ucsr/app/. Accesssed 1 June 2020].

[17] Higgins JPT, Eldridge S, Li T (2020). Chapter 23: Including variants on randomized trials. In: Higgins JPT, Thomas J, Chandler J, Cumpston M, Li T, Page MJ, Welch VA (editors). Cochrane Handbook for Systematic Reviews of Interventions version 6.1 (updated September 2020).

[18] Eldridge S, Campbell M, Campbell M, Drahota A, Giraudeau B, Higgins J, et al. Revised Cochrane risk of bias tool for randomized trials (RoB 2.0): Additional considerations for cluster-randomized trials October 2016 [Available from: https://www.riskofbias.info/welcome/rob-2-0-tool/archive-rob-2-0-cluster-randomized-trials-2016. Accessed: I June 2020].

[19] Husereau D, Drummond M, Petrou S, Carswell C, Moher D, Greenberg D (2013). Consolidated Health Economic Evaluation Reporting Standards (CHEERS) statement. BMJ.

[20] Abdurrahman ST, Lawson L, Blakiston M, Obasanya J, Yassin MA, Anderson RM (2017). Are patients with pulmonary tuberculosis who are identified through active case finding in the community different than those identified in healthcare facilities?. New microbes and new infections.

[21] den Boon S, Verver S, Lombard CJ, Bateman ED, Irusen EM, Enarson DA (2008). Comparison of symptoms and treatment outcomes between actively and passively detected tuberculosis cases: the additional value of active case finding. Epidemiol Infect.

[22] Shargie EB, Yassin MA, Lindtjorn B. (2006). Prevalence of smear-positive pulmonary tuberculosis in a rural district of Ethiopia. Int J Tuberc Lung Dis.

[23] Gopi PG, Chandrasekaran V, Narayanan PR. (2004). Failure to initiate treatment for tuberculosis patients diagnosed in a community survey and at health facilities under a DOTS programme in a district of South India. Indian J Tuberc.

[24] Balasubramanian R, Garg R, Santha T, Gopi PG, Subramani R, Chandrasekaran V (2004). Gender disparities in tuberculosis: report from a rural DOTS programme in south India. Int J Tuberc Lung Dis.

[25] Santha T, Renu G, Frieden TR, Subramani R, Gopi PG, Chandrasekaran V (2003). Are community surveys to detect tuberculosis in high prevalence areas useful? Results of a comparative study from Tiruvallur District, South India. Int J Tuberc Lung Dis.

[26] Harper I, Fryatt R, White A. (1996). Tuberculosis case finding in remote mountainous areas–are microscopy camps of any value? Experience from Nepal. Tuber Lung Dis.

[27] Cassels A, Heineman E, LeClerq S, Gurung PK, Rahut CB. (1982). Tuberculosis case-finding in Eastern Nepal. Tubercle.

[28] Shargie EB, Morkve O, Lindtjorn B. (2006). Tuberculosis case-finding through a village outreach programme in a rural setting in southern Ethiopia: community randomized trial. Bull World Health Organ.

[29] Shewade HD, Gupta V, Satyanarayana S, Kumar S, Pandey P, Bajpai UN (2019). Active versus passive case finding for tuberculosis in marginalised and vulnerable populations in India: comparison of treatment outcomes. Global health action.

[30] Shewade HD, Gupta V, Satyanarayana S, Pandey P, Bajpai UN, Tripathy JP (2019). Patient characteristics, health seeking and delays among new sputum smear positive TB patients identified through active case finding when compared to passive case finding in India. PloS one.

[31] Paiao DS, Lemos EF, Carbone AD, Sgarbi RV, Junior AL, da Silva FM (2016). Impact of mass-screening on tuberculosis incidence in a prospective cohort of Brazilian prisoners. BMC Infect Dis.

[32] Story A, Aldridge RW, Abubakar I, Stagg HR, Lipman M, Watson JM (2012). Active case finding for pulmonary tuberculosis using mobile digital chest radiography: an observational study. Int J Tuberc Lung Dis.

[33] Verver S, Bwire R, Borgdorff MW. (2001). Screening for pulmonary tuberculosis among immigrants: estimated effect on severity of disease and duration of infectiousness. Int J Tuberc Lung Dis.

[34] Churchyard GJ, Kleinschmidt I, Corbett EL, Murray J, Smit J, De Cock KM (2000). Factors associated with an increased case-fatality rate in HIV-infected and non-infected South African gold miners with pulmonary tuberculosis. Int J Tuberc Lung Dis.

[35] Capewell S, France AJ, Anderson M, Leitch AG (1986). The diagnosis and management of tuberculosis in common hostel dwellers. Tubercle.

[36] Jenum S, Selvam S, Jesuraj N, Ritz C, Hesseling AC, Cardenas V (2018). Incidence of tuberculosis and the influence of surveillance strategy on tuberculosis case-finding and all-cause mortality: a cluster randomised trial in Indian neonates vaccinated with BCG. BMJ Open Respir Res.

[37] Fox GJ, Nhung NV, Sy DN, Hoa NLP, Anh LTN, Anh NT (2018). Household-Contact Investigation for Detection of Tuberculosis in Vietnam. N Engl J Med.

[38] Muniyandi M, Thomas BE, Karikalan N, Kannan T, Rajendran K, Dolla CK (2020). Catastrophic costs due to tuberculosis in South India: comparison between active and passive case finding. Trans R Soc Trop Med Hyg.

[39] Gurung SC, Dixit K, Rai B, Caws M, Paudel PR, Dhital R (2019). The role of active case finding in reducing patient incurred catastrophic costs for tuberculosis in Nepal. Infect Dis Poverty.

[40] Hussain H, Mori AT, Khan AJ, Khowaja S, Creswell J, Tylleskar T (2019). The cost-effectiveness of incentive-based active case finding for tuberculosis (TB) control in the private sector Karachi. Pakistan. BMC Health Serv Res..

[41] Shewade HD, Gupta V, Satyanarayana S, Kharate A, Sahai KN, Murali L (2018). Active case finding among marginalised and vulnerable populations reduces catastrophic costs due to tuberculosis diagnosis. Global health action.

[42] Morishita F, Yadav RP, Eang MT, Saint S, Nishikiori N. (2016). Mitigating Financial Burden of Tuberculosis through Active Case Finding Targeting Household and Neighbourhood Contacts in Cambodia. PloS one.

[43] Sekandi JN, Dobbin K, Oloya J, Okwera A, Whalen CC, Corso PS. (2015). Cost-effectiveness analysis of community active case finding and household contact investigation for tuberculosis case detection in urban Africa. PloS one.

[44] World Health Organization. The End TB Strategy: global strategy and targets for tuberculosis prevention, care and control after 2015 2014 [Available from: https://www.who.int/tb/post2015_TBstrategy.pdf?ua=1. Acessed 1 October 2020].

[45] United Nations. Political declaration of the UN general assembly high-level meeting on the fight against tuberculosis 2018 [Available from: https://www.who.int/tb/unhlmonTBDeclaration.pdf Accessed: 1 October 2020.

[46] Nations United (2018). World Leaders Reaffirm Commitment to End Tuberculosis by 2030, as General Assembly Adopts Declaration Outlining Actions for Increased Financing. Treatment Access.

[47] McQuaid CF, Vassall A, Cohen T, Fiekert K, COVID/TB Modelling Working Group, White RG. The impact of COVID-19 on TB: a review of the data. IJTLD. 2021;In press(pre-print available at: https://theunion.org/sites/default/files/2021-03/0148_Review%20McQuiad%20V3.pdf). 10.5588/ijtld.21.0148 PMC8171247 34049605

[48] Oga-Omenka C, Tseja-Akinrin A, Boffa J, Heitkamp P, Pai M, Zarowsky C. (2021). Commentary: Lessons from the COVID-19 global health response to inform TB case finding. Healthc (Amst).

[49] MacPherson P, Houben RM, Glynn JR, Corbett EL, Kranzer K. (2014). Pre-treatment loss to follow-up in tuberculosis patients in low- and lower-middle-income countries and high-burden countries: a systematic review and meta-analysis. Bull World Health Organ.

[50] Churchyard GJ, Fielding K, Roux S, Corbett EL, Chaisson RE, De Cock KM, et al. Twelve-monthly versus six-monthly radiological screening for active case-finding of tuberculosis: a randomised controlled trial2011. 134-9 p. 10.1136/thx.2010.139048 21098802

[51] Gupta-Wright A, Corbett EL, van Oosterhout JJ, Wilson D, Grint D, Alufandika-Moyo M, et al. Rapid urine-based screening for tuberculosis in HIV-positive patients admitted to hospital in Africa (STAMP): a pragmatic, multicentre, parallel-group, double-blind, randomised controlled trial 2018. 292-301. 10.1016/S0140-6736(18)31267-4 PMC6078909 30032978

[52] Datiko DG, Lindtjorn B. (2009). Health extension workers improve tuberculosis case detection and treatment success in southern Ethiopia: a community randomized trial. PloS one.

[53] Marks GB, Nguyen NV, Nguyen PTB, Nguyen TA, Nguyen HB, Tran KH (2019). Community-wide Screening for Tuberculosis in a High-Prevalence Setting. N Engl J Med.

[54] Corbett EL, Bandason T, Duong T, Dauya E, Makamure B, Churchyard GJ (2010). Comparison of two active case-finding strategies for community-based diagnosis of symptomatic smear-positive tuberculosis and control of infectious tuberculosis in Harare, Zimbabwe (DETECTB): a cluster-randomised trial. Lancet.

[55] Marahatta SB, Yadav RK, Giri D, Lama S, Rijal KR, Mishra SR (2020). Barriers in the access, diagnosis and treatment completion for tuberculosis patients in central and western Nepal: A qualitative study among patients, community members and health care workers. PloS one.

[56] Aibana O, Dauria E, Kiriazova T, Makarenko O, Bachmaha M, Rybak N (2020). Patients' perspectives of tuberculosis treatment challenges and barriers to treatment adherence in Ukraine: a qualitative study. BMJ Open.

[57] Sullivan BJ, Esmaili BE, Cunningham CK. (2017). Barriers to initiating tuberculosis treatment in sub-Saharan Africa: a systematic review focused on children and youth. Global health action.

[58] Burke RM, Nliwasa M, Feasey HRA, Chaisson LH, Golub JE, Naufal F (2021). Community-based active case-finding interventions for tuberculosis: a systematic review. Lancet Public Health.

