# Supplementary material for: Does tuberculosis screening improve individual outcomes? A systematic review
Source: eClinicalMedicine. 2021 Sep 22;40:101127. doi: 10.1016/j.eclinm.2021.101127 (PMC8473670; doi:10.1016/j.eclinm.2021.101127)
Supplement: Supplementary file 1 [file mmc1.docx]

**Appendix 1 Search terms**

Search terms in EMBASE for each review are shown below. These were adapted for Pubmed/Medline, Scopus and the Cochrane Library

**Clinical review**

| 1 | 'tuberculosis'/exp OR 'lung tuberculosis'/exp |
| --- | --- |
| 2 | (‘tuberculosis’ OR ‘Pulmonary Consumption’ OR ‘Consumption, Pulmonary’ OR Phthisis OR ‘Tuberculoses’ OR “MDR-TB” OR “XDR-TB” OR “MDR TB” OR “XDR TB”):ab,ti,kw |
| 3 | 1 OR 2 |
| 4 | 'tuberculosis control'/exp OR 'case finding'/exp OR 'mass radiography'/exp OR 'mass screening'/exp OR 'contact examination'/exp OR 'screening'/exp |
| 5 | (‘Mass Chest X Ray’ OR ‘Mass Chest X-Rays’ OR ‘Screenings’ OR ‘screening’ OR ‘Cross-Sectional Studies’ OR ‘Case-detection’ OR ‘case finding’ OR ‘contact tracing’ OR ‘mass radiography’ OR ‘contact examination’ OR ‘health survey’ OR ‘cross-sectional’ OR 'prevalence survey' OR ‘prevalence studies’):ab,ti,kw |
| 6 | 4 OR 5 |
| 7 | 3 AND 6 |
| 8 | 'animal'/exp NOT ('animal'/exp AND 'human'/exp) |
| 9 | 7 NOT 8 |
| 10 | [1-11-2010]/sd |
| 11 | 9 AND 10 |

**Economic review**

| 1 | exp screening/ |
| --- | --- |
| 2 | exp case finding/ |
| 3 | exp mass radiography/ |
| 4 | exp tuberculosis control/ |
| 5 | exp contact examination/ |
| 6 | (screen* or case-find* or case-detect* or (active* adj3 case*) or (enhance* adj3 case*) or (intensi* adj3 case*) or (active* adj3 find*) or (enhance* adj3 find*) or (intensi* adj3 find*) or ACF or ECF or ICF).mp |
| 7 | or/1-6 |
| 8 | exp tuberculosis/ or exp lung tuberculosis/ |
| 9 | (tb or tuberculo*).mp |
| 10 | 8 or 9 |
| 11 | health economics/ |
| 12 | medical fee/ |
| 13 | exp economic evaluation/ |
| 14 | exp "health care cost"/ |
| 15 | (econom$ or cost or costs or costly or costing or price or prices or pricing or pharmacoeconomic$).ti,ab. |
| 16 | (expenditure$ not energy).ti,ab. |
| 17 | (value adj2 money).ti,ab. |
| 18 | budget$.ti,ab. |
| 19 | financ*.ti,ab. |
| 20 | (expense or expensive).ti,ab. |
| 21 | (pay or payment or payments or paid or paying).ti,ab. |
| 22 | (attention adj3 (pay or paid or paying)).ab. |
| 23 | 21 not 22 |
| 24 | ((spend or spending or spent) not ((spend or spending or spent) adj3 (time or hours))).ti,ab. |
| 25 | exp employment status/ |
| 26 | job security/ |
| 27 | unemploy*.ti,ab. |
| 28 | ((employ* or job or work*) adj1 (loss or lost or lose)).ti,ab. |
| 29 | ((employ* or job or work*) adj1 security).ti,ab. |
| 30 | redundan*.ti,ab. |
| 31 | ((productivity or productive) adj2 (loss or lost or lose)).ti,ab. |
| 32 | quality adjusted life year/ |
| 33 | life years.ti,ab. |
| 34 | or/11-20,23-33 |
| 35 | (rat or rats or mouse or mice or swine or porcine or murine or sheep or lambs or pigs or piglets or rabbit or rabbits or cat or cats or dog or dogs or cattle or bovine or monkey or monkeys or trout or marmoset$1).ti. and animal experiment/ |
| 36 | Animal experiment/ not (human experiment/ or human/) |
| 37 | 35 or 36 |
| 38 | 7 and 10 and 34 |
| 39 | 38 not 37 |
| 40 | limit 39 to yr="2010-2020" |
| 41 | limit 40 to (english or french or spanish) |
| 42 | remove duplicates from 41 |

**Appendix 2: Risk of bias assessment for studies identified in the clinical review**

**Section I. Risk of bias of observational studies reporting on smear grade or smear positivity among culture confirmed people with TB**

| **Study** | **Shewade 2019** | **Abdurrahman 2016** | **den Boon 2008** | **Santha 2003** | **Paiao 2016** | **Story 2012** | **Verver 2001** | **Capewell 1986** |
| --- | --- | --- | --- | --- | --- | --- | --- | --- |
| **Outcome** | **Smear grade** | | | | **Smear positivity among culture confirmed people with TB** | | | |
| **Study Design** | Cross-sectional | Cross-sectional | Cross-sectional | Cross-sectional | Cross-sectional data from cohort study | Cross-sectional | Cross-sectional | Cross-sectional |
| **Participant selection:** sample taking part similar to those not taking part / appropriate eligibility criteria | Unclear | Probably No | Probably No | Unclear | Probably Yes | Probably Yes | No | Unclear |
| **Exposure ascertainment:** objective data source | Yes | Yes | Yes | Probably Yes | Probably Yes | Probably No | Probably Yes | Unclear |
| **Outcome data source** | Treatment registers | Laboratory data from research | PCF: treatment register; screened: unclear | Treatment cards | Research records and clinical records/National notification data | National notification data | Anonymous database held by KNCV | Notification data |
| **Outcome ascertainment:** objective data source | Probably Yes | Yes | Unclear | Probably Yes | Probably Yes | Probably Yes | Probably Yes | Probably Yes |
| **Analysis:** adequate control for confounders | No | No | No | No | No | Probably No* | No | No |

PCF=passive case-finding; KNCV=Dutch tuberculosis foundation; *undertook and adjusted analysis but residual confounding is possible

**Section II. Observational studies reporting on treatment outcomes and case fatality**

| **Study** | **Shewade 2019** | **den Boon 2008** | **Santha 2003** | **Verver 2001** | **Churchyard 2000** | **Harper 1996** | **Cassels 1982** |
| --- | --- | --- | --- | --- | --- | --- | --- |
| **Study Design** | Cross-sectional | Cross- sectional | Cross -sectional | Cross -sectional | Cross-sectional data from cohort study | Cross-sectional | Cross-sectional |
| **Participant selection:** sample taking part similar to those not taking part / appropriate eligibility criteria | Unclear | Probably No | Unclear | No | Unclear | Unclear | Probably Yes |
| **Exposure ascertainment:** objective data source | Yes | Yes | Probably Yes | Probably Yes | Unclear | Unclear | Unclear |
| **Outcome data source** | Treatment registers | PCF: treatment register; screened: not specified | Treatment cards | Anonymous database held by KNCV | Limited autopsy and clinical records | Clinical records | Likely mix of clinical records and follow-up but not specified |
| **Outcome ascertainment:** objective data source | Probably Yes | Unclear | Probably Yes | Probably Yes | Probably Yes | Probably Yes | Unclear |
| **Analysis:** adequate control for confounders | Probable No* | No | No | No | Probably No* | No | No |

PCF=passive case-finding; KNCV=Dutch tuberculosis foundation; *undertook an adjusted analysis but residual confounding is possible

**Section III. Observational studies reporting on pre-treatment loss to follow-up and time to first contact with health services, diagnosis and treatment start**

| **Item** | **Gopi 2005** | **Balasumbramanian 2004** | **Santha 2003** | **Shewade 2019** | **Abdurrahman 2016** | **Verver 2001** | **Shargie 2006** |
| --- | --- | --- | --- | --- | --- | --- | --- |
| **Study Design** | Cross-sectional | Cross-sectional | Cross-sectional | Cross-sectional | Cross-sectional | Cross-sectional | Cross-sectional |
| **Participant selection:** sample taking part similar to those not taking part / appropriate eligibility criteria | Probably Yes | Unclear | Unclear | Probably No | Probably No | No | Probably No |
| **Exposure ascertainment:** objective data source | Unclear | Unclear | Probably Yes | Yes | Yes | Probably Yes | Yes |
| **Outcome data source** | Insufficient information | Insufficient information | Self-report | Self-report and clinical records | Self-report | Self-report | Self-report |
| **Outcome ascertainment:** objective data source | Unclear | Unclear | No | Probably No | No | No | No |
| **Analysis:** adequate control for confounders | No | No | No | Probably No* | No | No | No |

*undertook an adjusted analysis but residual confounding is possible

**Section IV: Cluster randomised controlled trials reporting on treatment outcomes and mortality (risk of bias assessed using the Cochrane Risk of Bias assessment tool 2.0)**

|  | **Study** | | |
| --- | --- | --- | --- |
|  | **Shargie 2006** | **Jenum 2018** | **Fox 2018** |
| **Outcome assessed** | Treatment success  Case fatality | All-cause mortality | All-cause mortality |
| **Risk of bias domains** |  |  |  |
| Bias arising from the randomisation process | Some concerns | Low | Low |
| Bias arising from the timing of identification and recruitment of individual participants in relation to the timing of randomisation | Low | Low | Low |
| Bias due to deviation from the intended interventions | Low | Low | Low |
| Bias due to missing outcome data | Low | High | Low |
| Bias in measurement of the outcome | Low | Low | Low |
| Bias in the selection of the reported results | Low | Low | Some concerns |
| **Risk of bias judgement** | **Some concern** | **High** | **Some concern** |
| Comments | Insufficient information to assess one bias domain | Bias due to missing outcome data may have underestimated deaths, with proportionally less deaths reported among the passive case-finding group. Therefore the effect of screening on mortality could be a minimum estimate | All-cause mortality was a post-hoc analysis |

**Appendix 3:** Smear grade 3+ and 2+ among all smear positive TB patients in n=3 general-population observational studies

| **First author, country, screening tool** | **Group** | **Smear grade (3+ and 2+) / all smear positives** | | **Comments** |
| --- | --- | --- | --- | --- |
|  |  | **n/N*** | **% (95%CI)** |  |
| Abdurrahman 2016 Nigeria  Symptoms | Screen | 268/480 | 56% (51-60%) | Diagnosed TB patients  Screened vs PCF - screened group more likely to be older, married and less likely to be HIV infected. |
|  | PCF | 151/208 | 73% (66-79%) |  |
| den Boon 2008 South Africa  Smear & culture | Screen | 10/18 | 56% (31-78%) | Denominator for smear grade - screened group includes those lost to follow-up pre-treatment; PCF those starting treatment only  Diagnosed in screened and on treatment in PCF groups - no difference in age and gender. |
|  | PCF | 314/446 | 70% (66-75%) |  |
| Santha 2003 India  CXR and symptoms | Screen | 39/96 | 41% (31-51%) | Denominator for smear grade - screened group includes those lost to follow-up pre-treatment; PCF those starting treatment only  All (smear +ve and -ve) diagnosed in screened and on treatment in PCF groups - screened group more likely to be older, male, illiterate, sole earner, have poor quality house and a 1 room house |
|  | PCF | 228/330 | 69% (64-74%) |  |

*n/N=number with smear grade (3+ and 2+)/total number with smear grade scanty, 1+, 2+ and 3+; 95%CI = 95% confidence interval; PCF=passive case-finding; CXR=chest radiograph;

Data from *Shewade HD et al. Active versus passive case finding for tuberculosis in marginalised and vulnerable populations in India: comparison of treatment outcomes. Global health action. 2019;12(1):1656451* not included as smear data only provided as scanty/1+/2+ and 3+, and therefore could not be recategorized.

**Appendix 4: Risk of bias assessment for studies identified in the economics review using the CHEERS checklist**

| Questions | Responses | | | | | |
| --- | --- | --- | --- | --- | --- | --- |
|  | **Muniyandi**  **2020** | **Gurung 2019** | **Hussain 2019** | **Shewade 2018** | **Morishita 2016** | **Sekandi**  **2015** |
| Does the title include economic evaluation terms as ‘‘cost" or "cost-effectiveness’’ and describe the interventions compared? | Yes | Yes | Yes | Yes | Yes | Yes |
| Does the abstract provide a structured summary of objectives, perspective, setting, methods, results and conclusions? | Yes | Yes | Yes | Yes | Yes | Yes |
| Does the introduction include an explicit statement of the broader context for the study and present the study question and its relevance for health policy or practice decisions? | Yes | Yes | Yes | Yes | Yes | Yes |
| Is the study population clearly described? | Yes | Yes | Yes | Yes | Yes | Yes |
| Are competing alternatives clearly described? | Yes | Yes | Yes | Yes | Yes | Yes |
| Is a well-defined research question posed in answerable form? | Yes | Yes | Yes | Yes | Yes | Yes |
| Is the economic study design appropriate to the stated objective? | Yes | Yes | Yes | Yes | Yes | Yes |
| Is the chosen time horizon appropriate in order to include relevant costs and consequences? | N/A | N/A | yes | N/A | N/A | Yes |
| Is the actual perspective chosen appropriate? | Yes | Yes | Yes | Yes | Yes | Yes |
| Are all important and relevant costs for each alternative identified? | Yes | Yes | Yes | Yes | Yes | Yes |
| Are all costs measured appropriately in physical units? | Yes | Yes | Yes | Yes | Yes | Yes |
| Are costs valued appropriately? | Yes | Yes | Yes | Yes | Yes | Yes |
| Are all important and relevant outcomes for each alternative identified? | N/A | N/A | Yes | N/A | N/A | Yes |
| Are all outcomes measured appropriately in physical units? | N/A | N/A | Yes | N/A | N/A | Yes |
| Are outcomes valued appropriately? | N/A | N/A | Yes | N/A | N/A | Yes |
| Is an incremental analysis of costs and outcomes of alternatives performed? | N/A | N/A | Yes | N/A | N/A | Yes |
| Are all future costs and outcomes discounted appropriately? | N/A | N/A | Yes | N/A | N/A | Yes |
| Are all important variables, whose values are uncertain, appropriately subjected to sensitivity analysis? | N/A | N/A | Yes | N/A | N/A | Yes |
| Does the study discuss the generalizability of the results to other settings and patient/client groups? | Yes | Yes | Yes | No | Yes | Yes |
| Do the conclusions follow from the data reported? | Yes | Yes | Yes | Yes | Yes | Yes |
| Does the article indicate that there is no potential conflict of interest of study researcher(s) and funder(s)? | Yes | Yes | Yes | Yes | No* | No* |

**Sometimes not indicated in the manuscript but submitted through the online submission system*

**Appendix 5: Pre-treatment and treatment costs for n=3 studies separating costs**

| **First author, population and screening method, illness period and costs reported** | | **Total diagnosis/pre-treatment costs** | | | **Total treatment costs** | | | **Comments** |
| --- | --- | --- | --- | --- | --- | --- | --- | --- |
|  |  | **Screened** | **PCF** | **p-value** | **Screened** | **PCF** | **p-value** |  |
| **Muniyandi (2020)**; India  General population; symptoms and CXR screen  Diagnosis and treatment  Direct (medical and non-medical) and indirect costs  N=110 in screened and N=226 in PCF group | Mean  (SEM) | 30  (10) | 130  (13) | 0.001 | 39  (14) | 97  (12) | 0.004 | Mean diagnosis costs – screened vs PCF  Direct: 16 vs 75; p=0.001  Indirect: 14 vs 55; p=0.001  Mean treatment costs – screened vs PCF  Direct: 2 vs 4; p=0.027  Indirect: 37 vs 93; p=0.001 |
| **Gurung (2019);** Nepal  OPD attendees, social contacts of people with TB, general population TB camps; symptom screen  Pre-treatment (from symptom start) and intensive treatment phase  Direct (medical and non-medical) and indirect costs  N=50 in screened and N=49 in PCF group | Median (IQR) | 132  (23–258) | 172  (60–405) | 0.103 | 85  (56-144) | 104  (45-193) | 0.557 | Median (IQR) pre-treatment costs – screened vs PCF  Direct medical: 14 (4-28) vs 32 (11-79); p=0.001  Direct non-medical: 3 (2-10) vs 10 (3-38); p=0.004  Indirect: 63 (5-255) vs 43 (14-248); p=0.430  Median (IQR) treatment costs – screened vs PCF  Direct medical: p=0.070*  Direct non-medical: 0 (0-14) vs 1.3 (0-45); p=0.034  Indirect: 55 (30-96) vs 60 (35-83)’ p=0.817 |
| **Morishita (2016);** Cambodia  HH and neighbourhood contacts; CXR screen  Pre-treatment and during 6 months of treatment  Direct (medical and non-medical) and indirect costs  N=108 in screened and N=100 in PCF group | Median (IQR) | 5  (1-26) | 22  (4-71) | <0.001 | 233  (52-568) | 235  (88-636) | 0.367 | Median (IQR) pre-treatment costs – screened vs PCF  Direct: 2 (1-11) vs 15 (2-47); p<0.001  Indirect: 0 (0-4) vs 1 (0-4); p=0.073  Median (IQR) treatment costs – screened vs PCF  Direct: 67 (22-123) vs 90 (45-202); p=0.014  Indirect: 85 (0-450) vs 60 (0-382); p=0.553 |

All values (costs and proportions) rounded to the nearest whole number; PCF=passive case-finding; CXR=chest radiograph; SEM=standard error of the mean; OPD=outpatient department; IQR=interquartile range; HH=household; *comparing no costs incurred in screened group vs costs incurred in PCF group (for medicines)
